# Development of nanobody-conjugated LL37 for synergistic therapy against MDR *Acinetobacter baumannii*

**DOI:** 10.1128/msphere.00779-25

**Published:** 2026-02-06

**Authors:** Apisitt Thaiprayoon, Worrapoj Oonanant, Siriphan Boonsilp, Nonth Submunkongtawee, Phoomintara Longsompurana, Duangtip Moonmangmee, Pinpunya Riangrungroj, Jeerapond Leelawattanachai, Lueacha Tabtimmai, Andrew C. Kruse, Matthew P. DeLisa, Phattara-orn Havanapan, Dujduan Waraho-Zhmayev

**Affiliations:** 1 Biological Engineering Program, Faculty of Engineering, King Mongkut’s University of Technology Thonburi 124260 , Bangkok, Thailand; 2 Department of Basic Medical Science, Faculty of Medicine Vajira Hospital, Navamindradhiraj University 292577, Bangkok, Thailand; 3 Department of Clinical Pathology, Faculty of Medicine Vajira Hospital, Navamindradhiraj University 292577, Bangkok, Thailand; 4 Department of Microbiology, Faculty of Science, King Mongkut’s University of Technology Thonburi 562724 , Bangkok, Thailand; 5 National Center for Genetic Engineering and Biotechnology (BIOTEC), National Science and Technology Development Agency (NSTDA), Khlong Nueng, Pathum Thani, Thailand; 6 National Nanotechnology Center (NANOTEC), National Science and Technology Development Agency (NSTDA), Khlong Luang, Pathum Thani, Thailand; 7 Department of Biotechnology, Faculty of Applied Science, King Mongkut’s University of Technology North Bangkok 562650 , Bangkok, Thailand; 8 Food and Agro-Industrial Research Center, King Mongkut's University of Technology North Bangkok 67994, Bangkok, Thailand; 9 Department of Biological Chemistry and Molecular Pharmacology, Harvard Medical School 189702 , Boston, Massachusetts, USA; 10 Robert F. Smith School of Chemical and Biomolecular Engineering, Cornell University 251794, Ithaca, New York, USA; 11 Cornell Institute of Biotechnology, Cornell University 635264, Ithaca, New York, USA; 12 Institute of Molecular Biosciences, Mahidol University 98841, Salaya, Nakhon Pathom, Thailand; University of Nebraska Medical Center College of Medicine, Omaha, Nebraska, USA

**Keywords:** *Acinetobacter baumannii*, outer membrane protein A, LL37, conjugated nanobody, nanobody, drug-resistance bacteria, MACS-based yeast display

## Abstract

Multidrug resistance (MDR) of the pathogen *Acinetobacter baumannii* is a major challenge to global healthcare due to the limited treatment options. The emergence of MDR bacteria necessitates innovative therapeutic approaches, especially given the associated economic burden and the rapid spread of infections. Conventional treatments such as antibiotics and vaccines face significant obstacles. Antimicrobial peptides (AMPs) such as LL37 have potential as an alternative treatment due to their broad-spectrum activity and ability to target specific bacterial structures such as the outer membrane protein A (OmpA). The efficacy of AMPs can be enhanced by using nanobodies (Nbs) that bind to bacterial OmpA, guiding LL37 precisely to its target. In this study, *A. baumannii* OmpA (AbOmpA)-specific Nbs (NbO7 and NbO13) were efficiently isolated through magnetic-activated cell sorting-based screening of a yeast surface display library, eliminating the need for specialized equipment. Nbs exhibited specific, dose-dependent binding to the target. Conjugation of Nbs with LL37 effectively inhibited the growth of MDR *A. baumannii*. This approach leverages the natural antimicrobial properties of AMPs and enhances their specificity and effectiveness by targeting bacterial cell surface proteins. LL37-conjugated AbOmpA-Nbs present a promising therapeutic strategy against MDR *A. baumannii* and other resistant pathogens.

**IMPORTANCE:**

Multidrug-resistant (MDR) *Acinetobacter baumannii* poses a major global health threat due to its resistance to nearly all available antibiotics and its persistence in hospital settings. This challenge underscores the urgent need for new therapeutic approaches beyond conventional drugs. In this study, we developed an innovative strategy that combines the human antimicrobial peptide LL37 with nanobodies (Nbs) targeting the outer membrane protein A (OmpA), a key virulence and survival factor of *A. baumannii*. OmpA-specific Nbs were efficiently isolated from a fully synthetic library using a simple, low-cost selection approach without animal immunization. When conjugated with LL37, these Nbs bound specifically to OmpA and strongly inhibited MDR *A. baumannii* growth *in vitro*. Our findings introduce a simple yet powerful platform for generating targeted Nb-peptide conjugates, offering strong potential for adaptation against other antibiotic-resistant pathogens and contributing to the development of next-generation biologics to overcome antibiotic limitations.

## INTRODUCTION

*Acinetobacter baumannii* is a gram-negative opportunistic bacterium that has been identified as one of the “ESKAPE” pathogens. Currently, an estimated 742 million USD is spent annually combatting multidrug-resistant (MDR) pathogens (1, 2). The bacteria are resistant to almost all currently available antibiotics, thus limiting effective treatment options (3). There have been reports of drug resistance in *A. baumannii* to aminoglycosides, tetracyclines, fluoroquinolones, chloramphenicol, tigecycline, and colistin. Genomic studies have demonstrated that *A. baumannii* utilizes a complex virulence strategy involving 16 gene islands that contribute to its pathogenicity (4). The global prevalence of MDR *A. baumannii* is rising, especially in regions with inadequate infection control practices, such as developing countries in Southeast Asia (5–8). Key mechanisms involved in bacterial virulence include efflux pumps, altered porins, β-lactamase production, biofilm formation, and horizontal gene transfer (1, 2). Several attempts have been made to identify new approaches against these MDR infections; these include the development of new antibiotics and vaccines (9–12). Antibiotics may have side effects that destroy the patient’s normal microflora (13), while vaccines can enhance the immune system to directly resist infection. Thus, research efforts have focused on selecting antigens from *A. baumannii* for vaccine development. The outer membrane protein A (OmpA) is the most abundant surface-exposed protein of the bacterium (14); it is produced throughout the bacterial lifespan and plays a crucial role in cell survival. For example, the survival rate of OmpA-vaccinated mice increased by 50%–80% after challenge with MDR *A. baumannii* (15). However, despite the advantages of vaccines and their prevention of infection, the immune system response is limited, as it takes at least 2 to 3 weeks to produce effective antibodies against the pathogen, while MDR *A. baumannii* can multiply and cause several severe symptoms within 1 week. Antimicrobial peptides (AMPs) occur naturally in many organisms, and they play a crucial role in innate immunity. AMPs have been engineered to enhance their stability, activity, or specificity, thereby allowing for targeted therapies and customization for specific infections (16–18).

In 1989, a new type of antibody was identified from the serum of dromedary species. These antibodies were unique in that they lacked the light chain and the first constant heavy domain (CH1) and were thus named heavy-chain IgG. The heavy-chain variable domain of heavy-chain antibodies (VHH), or “nanobody” (Nb), is a fragment consisting of only a single monomeric variable antibody domain, making it the smallest antibody fragment that can specifically bind to an antigen (19, 20). Compared to traditional antibodies, Nbs have several advantages, including their small size that supports binding to concave epitopes, tolerance to a wide range of temperatures and environments, ease of renaturing, and high expression levels in the *Escherichia coli* expression system (21–23). These Nbs have applications in various fields, including immunoprecipitation, chaperone-assisted crystallization, gene activation or inactivation, protein-protein interactions, cellular bioimaging, *in vivo* and *in vitro* disease diagnosis, and the development of therapeutics (24, 25). Here, we aimed to screen *A. baumannii* OmpA (AbOmpA)-specific Nbs to construct AMP-conjugated Nbs as a new therapeutic approach for the treatment of MDR *A. baumannii*. LL37 is a part of the human AMP known as cathelicidin (hCAP-18) that exhibits broad-spectrum antimicrobial activity against various pathogens, including gram-positive and gram-negative bacteria, fungi, and viruses (26–28). The peptide is a lysine- and histidine-rich helical protein related to bacterial cell membrane binding that causes membrane disruption and hence cell death. Previously, AbOmpA was demonstrated to be strongly targeted by LL37, where a low concentration of LL37 could hinder bacterial growth (29–31). Since Nbs can interact with hidden epitopes, we hypothesized that Nb could direct LL37 to bind to the OmpA, facilitating synergistic inhibition of MDR *A. baumannii*. These newly LL37-conjugated AbOmpA-Nbs hold promise for their ability to target AbOmpA, thereby establishing a new therapeutic biomolecule against *A. baumannii* infection.

## RESULTS

### AbOmpA structural and amino acid sequence similarities among drug-resistant *A. baumannii* isolated from a hospital in Thailand

**Fig 1:**
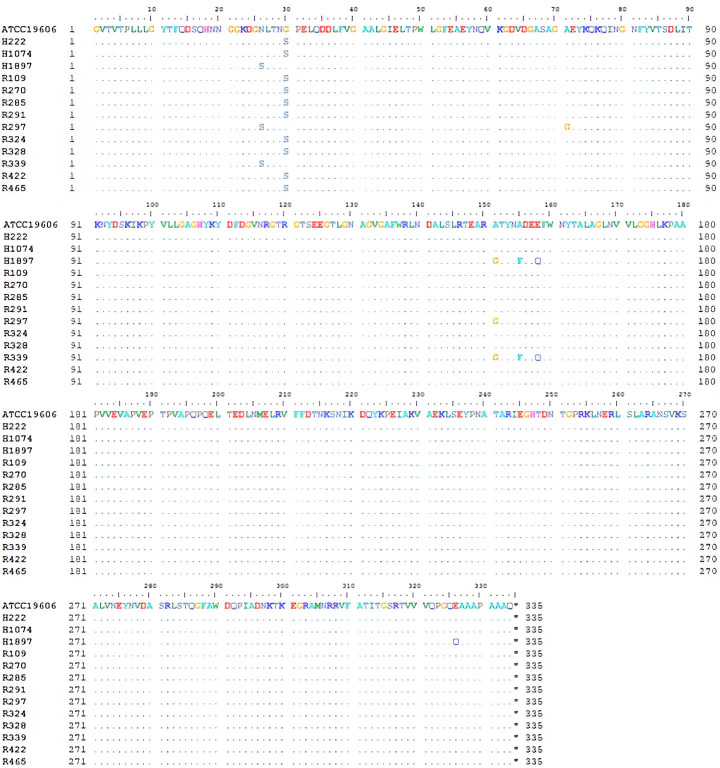
Comparison of amino acid residues of AbOmpA between a non-MDR strain of *A. baumannii* and 13 MDR clinical isolates collected from Vajira Hospital, Thailand. The AbOmpA genes from all 14 isolates were amplified using PCR and subsequently analyzed by DNA sequencing. The obtained gene sequences were translated into amino acid sequences and compared using BioEdit software.

The AbOmpA protein consists of two key structural regions. The first is an N-terminal domain, a region with an antiparallel β-barrel structure composed of eight transmembrane strands that integrate into the outer membrane. These eight strands are connected by four long loops that extend across the surface of the outer membrane. The second region is the periplasmic C-terminal domain, featuring three short turns that fold into a compact, globular structure located within the periplasmic space (17, 32). To assess the similarity of amino acid sequences, the AbOmpA gene fragments from several MDR *Acinetobacter baumannii* isolates were subjected to Sanger sequencing. Figure 1 illustrates the alignment of the AbOmpA amino acid residues (344 residues), revealing the similarities between the non-MDR *A. baumannii* ATCC19606 and 13 MDR *A. baumannii* isolates. There were seven amino acid variants, six of which were distributed in the N-terminal domain, and one located in the periplasmic C-terminal domain. An investigation of the motif scan also showed that AbOmpA from all 14 isolates contained similar motifs, an outer membrane β-barrel (residues 9–169), an OprF membrane domain (residues 33–174), and an outer membrane A motif (residues 211–307). In comparison, most of the differences in the amino acid sequences were in the position of the N-terminal domain, while the C-terminal contained the conservative amino acid residues among *A. baumannii* isolates (Fig. 1).

### Screening and characterization of Nbs specific to AbOmpA (*A. baumannii* ATCC19606)

**Fig 2:**
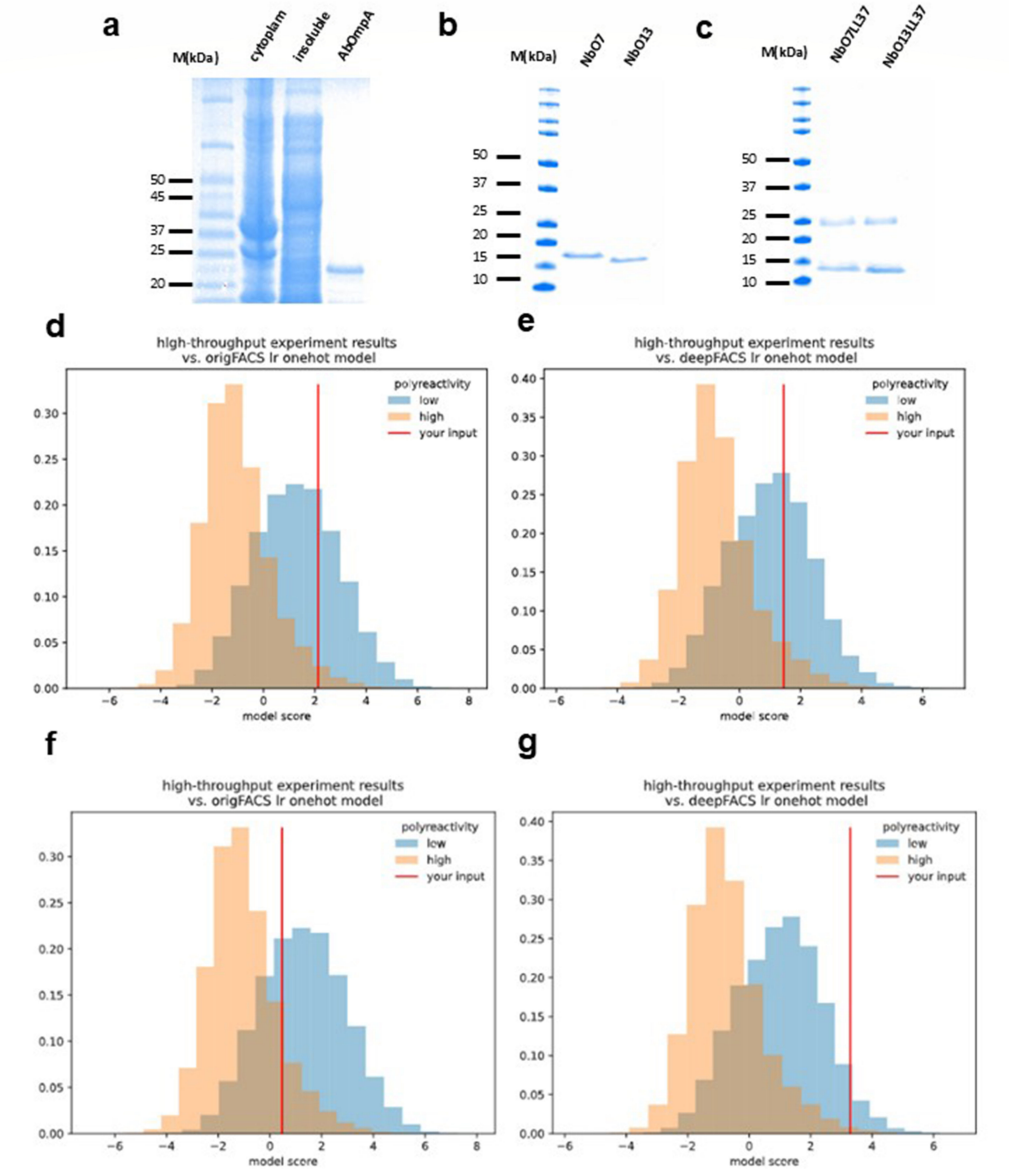
Expression and binding activity of FLI-TRAP-selected Nb clones. SDS-PAGE analysis of purification of AbOmpA (**a**), AbOmpA-specific Nbs NbO7 and NbO13 (**b**), and LL37-conjugated Nbs of NbO7LL37 and NbO13LL37 (**c**) following purification by Ni-NTA column chromatography. (**d, e**) The origFACS lr one-hot model and deepFACS lr one-hot model of NbO7. (**f, g**) The origFACS lr one-hot model and deepFACS lr one-hot model of NbO13 from one-hot and three-mer logistic regression models.

According to previous reports, AbOmpA is a critical virulence factor that plays a key role in the survival mechanisms of *A. baumannii*, including biotic and abiotic adhesion, biofilm formation, eukaryotic cell infection, antibiotic resistance, and immune evasion (32–34). Due to its high antigenicity, AbOmpA is readily recognized by the human humoral immune response (35, 36). Additionally, AbOmpA is expressed at every stage of the life cycle of *A. baumannii*, suggesting that it may be an ideal target for drug development (17). In this study, we aimed to identify specific Nbs targeting the N-terminal region of AbOmpA (NT-AbOmpA) that spans residues 1–135 and comprises a characteristic eight β-barrel structure. These Nbs could provide a novel therapeutic strategy for targeting *A. baumannii*. To achieve this, we purified the N-terminus of AbOmpA (Fig. 2a) and utilized magnetically activated cell sorting (MACS) to screen for NT-AbOmpA-specific Nbs from a synthetic yeast display library. The NT-AbOmpA protein (ATCC19606) was expressed in an *E. coli* system and purified through Ni-NTA column chromatography to ensure high purity for MACS screening (Fig. 2b).

**Fig 3:**
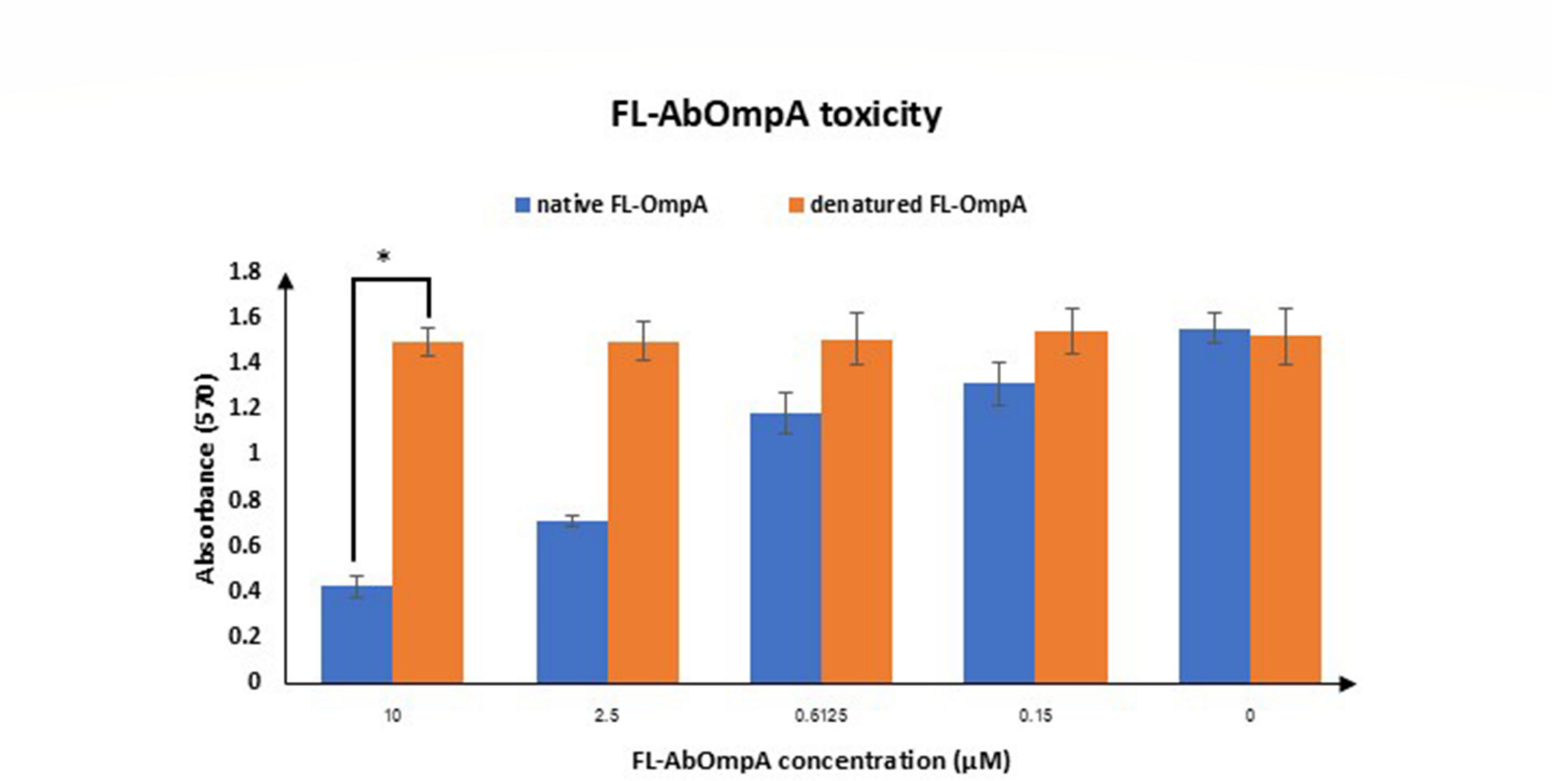
L929 fibroblast cells were treated with refolded and denatured FL-AbOmpA at various concentrations (0 µM–10 µM) for 24 h to assess cytotoxicity. Refolded FL-AbOmpA induced a dose-dependent reduction in cell viability, whereas denatured FL-AbOmpA showed no significant cytotoxic effect. These results indicate that the refolded FL-AbOmpA retains its native-like conformation and functional activity, consistent with the presence of a biologically active C-terminal domain responsible for cytotoxicity. Data are presented as mean ± SD from three independent experiments. Statistical significance was determined using a paired Student’s *t*-test. *P < 0.05.

To assess whether refolded full-length AbOmpA (FL-AbOmpA) regained its native conformation, both refolded and denatured (boiled for 10 min) forms of the protein were incubated with L929 cells in 96-well plates. The C-terminal region of AbOmpA naturally contains a nuclear localization signal motif known to exert cytotoxic effects on host cells. MTT assay results showed that the refolded AbOmpA induced significant growth inhibition of L929 cells at concentrations of 10, 2.5, and 0.6125 µM, whereas the denatured AbOmpA did not cause any detectable cytotoxicity (Fig. 3). These findings suggest that the refolded FL-AbOmpA retained its correct tertiary structure, enabling it to exhibit the characteristic cytotoxic property associated with the native protein.

To further assess the polyreactivity of these Nbs, computational predictions were conducted using one-hot and 3-mer logistic regression models. In Fig. 2d through g, the blue areas in the origFACs and deepFACs lr one-hot models indicate low polyreactivity, while the orange areas correspond to high polyreactivity (37). The predictions of NbO7 and NbO13 were as follows: origFACs lr one-hot scores of 2.05 and 0.53 and deepFACs lr one-hot scores of 0.84 and 3.42, respectively. Both the origFACs and deepFACs lr one-hot models indicated that both Nbs exhibited low polyreactivity. This low polyreactivity suggests that both NbO7 and NbO13 are specific to NT-AbOmpA and unlikely to bind non-specifically to other targets, an essential feature for their potential therapeutic use.

**Fig 4:**
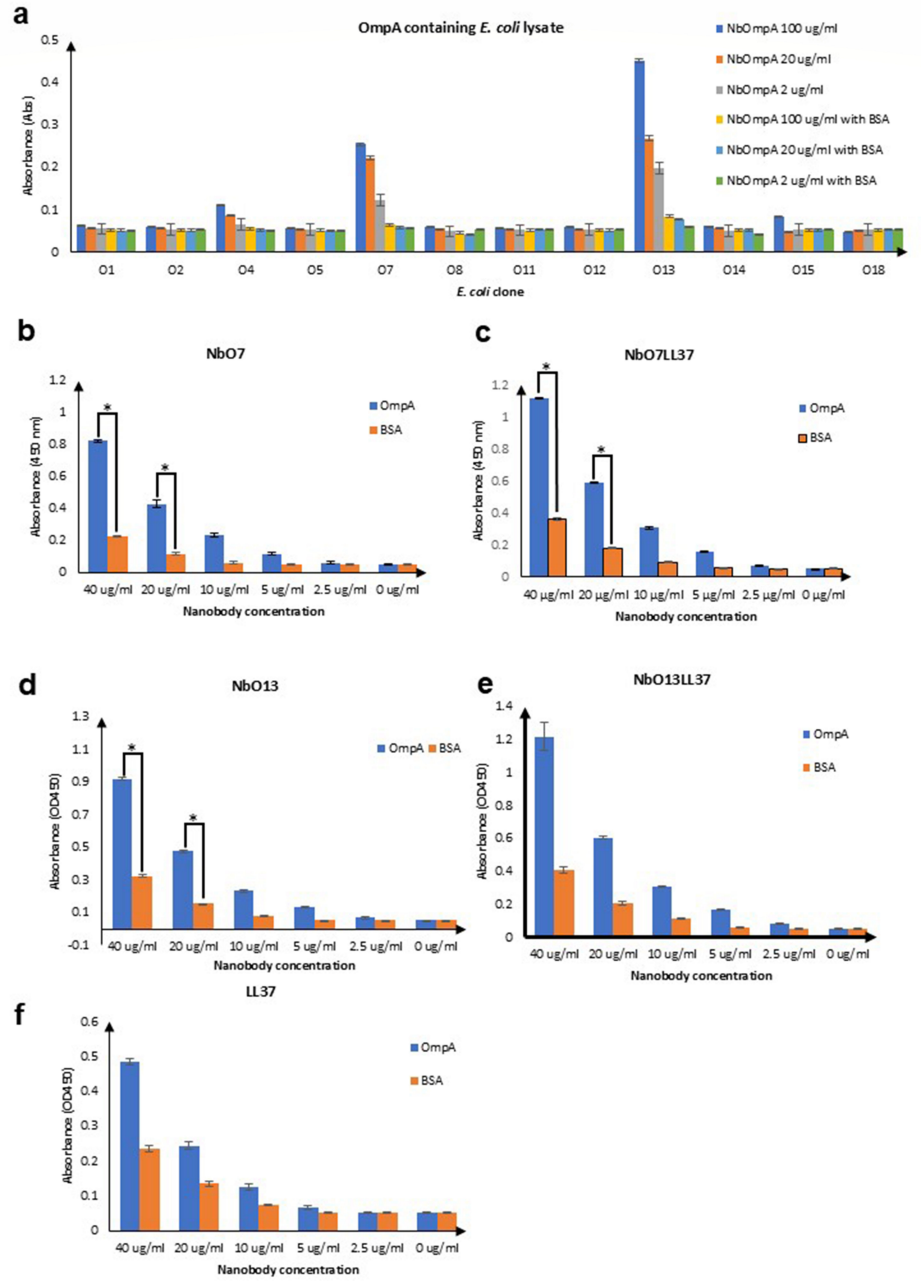
Detailed binding characteristics of AbOmpA-specific Nbs with NT-AbOmpA. (**a**) ELISA analysis of AbOmpA-specific Nbs from yeast display screening with AbOmpA as an immobilized antigen. (**b–f**) ELISA analyses of NbO7, NbO13, NbO7LL37, and NbO13LL37 with purified NT-AbOmpA. BSA served as negative control (orange bars). Data are the averages of three biological replicates and are shown as mean ± standard deviation. Statistical significance was determined using Student’s *t*-test to indicate statistically significant differences (*P < 0.05).

**Fig 5:**
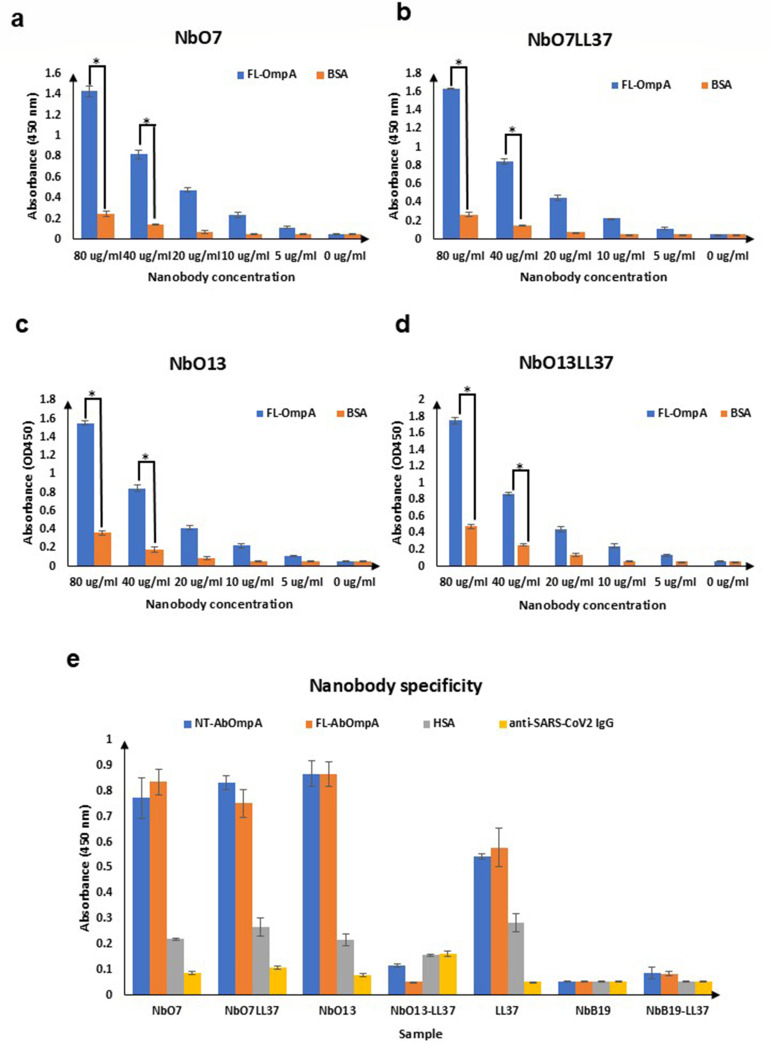
Detailed binding characteristics of AbOmpA-specific Nbs with FL-AbOmpA. (**a–d**) ELISA analyses of NbO7, NbO13, NbO7LL37, and NbO13LL37 with purified FL-AbOmpA. (**e**) ELISA analysis demonstrates the specificity of AbOmpA-specific Nbs from yeast display screening. Strong binding was observed toward NT-AbOmpA and FL-AbOmpA, whereas low binding signals were detected for non-related antigens, HSA and anti-SARS-CoV-2 IgG. Data are the averages of three biological replicates and are shown as mean ± standard deviation. Statistical significance was determined using Student’s *t*-test to indicate statistically significant differences (*P < 0.05).

The binding activities of the selected Nbs against NT-AbOmpA were compared using an ELISA assay. NbO13 demonstrated the highest binding activity, with an OD_450_ value of 0.4, indicating a strong affinity for NT-AbOmpA. In contrast, NbO7 showed slightly lower binding activity, with an OD_450_ of 0.3, suggesting a weaker interaction but still a notable affinity for NT-AbOmpA (Fig. 4a). To characterize the binding activities of these Nbs, purified NbO7 and NbO13 were tested in ELISA assays against three strains of *A. baumannii* (ATCC19606, R328, and R422), and the results showed dose-dependent binding against all three strains of NT-AbOmpA. The high specificity and low polyreactivity of these Nbs support the potential of NbO7 and NbO13 as tools for modulating the virulence of *A. baumannii* and thus suggesting them as candidates for further development in therapeutic applications targeting *A. baumannii* infections (Fig. 4b through f). In addition, the binding activities of NbO7, NbO13, NbO7LL37, and NbO13LL37 were further examined against FL-AbOmpA (Fig. 5a through d). The results obtained from ELISA assays using both NT-AbOmpA and FL-AbOmpA showed consistent binding patterns, implying that the Nbs recognize conserved epitopes within the AbOmpA protein. This concordance suggests that the Nbs are likely target-specific antigenic regions that are preserved between the N-terminal domain and the full-length form of AbOmpA. Moreover, Fig. 5e demonstrates that NbO7, NbO13, NbO7LL37, and NbO13LL37 bind specifically to both NT-AbOmpA and FL-AbOmpA, with markedly higher binding signals compared to unrelated antigens, including HSA and anti-SARS-CoV-2 IgG. These results further support the notion that the selected Nbs exhibit high specificity toward OmpA. It is plausible that the Nbs isolated through yeast display recognize distinct epitopes located within the N-terminal region of AbOmpA, which are preserved in the full-length protein and are likely critical for mediating their strong and specific binding. This specificity may arise from structural features unique to the N-terminal domain, allowing the Nbs to discriminate AbOmpA from other unrelated proteins.

### 3D model reveals the interactions of NbO7 and NbO13 with AbOmpA

**Fig 6:**
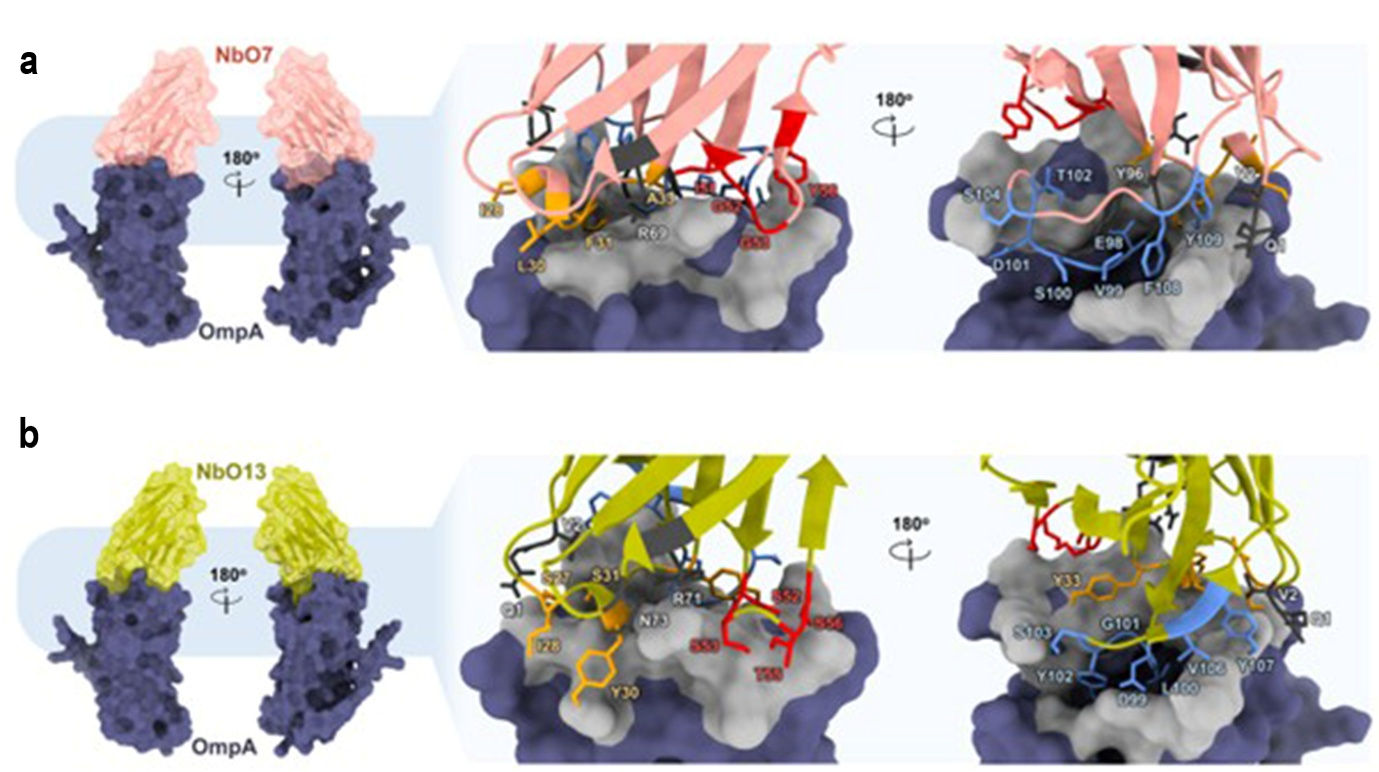
Predicted amino acid interactions between the isolated Nbs and AbOmpA. The interactions of (**a**) NbO7 and (**b**) NbO13 with AbOmpA.

**Fig 7:**
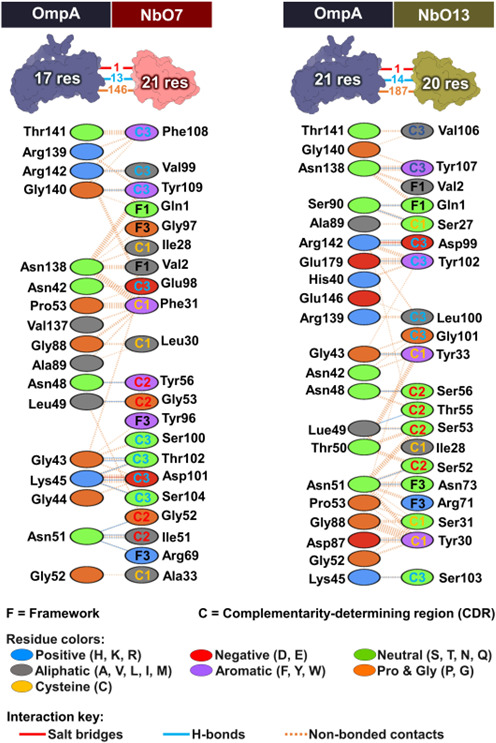
Protein-protein interactions of the wt PCSK9 with NbO7 and NbO13.

We performed a molecular docking analysis using the ClusPro server to explore how NbO7 and NbO13 might interact with NT-AbOmpA. The analysis revealed that NbO7 is predicted to form a total of 27 interactions with NT-AbOmpA, consisting of 1 electrostatic interaction, 6 hydrophobic interactions, and 20 hydrogen bonds. Similarly, NbO13 formed 18 interactions, 1 electrostatic interaction and 17 hydrogen bonds. Interestingly, both Nbs targeted the flexible loop regions of the NT-AbOmpA that are exposed to the external environment (Fig. 6 and 7). These flexible loops are critical structural features that could facilitate the binding of Nbs, making them attractive targets. According to the molecular docking model, both NbO7 and NbO13 possibly interacted with highly conserved regions of NT-AbOmpA that were shared across all 14 *A*. *baumannii* isolates. This observation is significant, as it suggests that the binding sites of NbO7 and NbO13 are not only conserved within a specific strain but also across a diverse set of *A. baumannii* strains. This conservation across isolates implies that these Nbs may interact with non-MDR and MDR forms of NT-AbOmpA, thereby offering a broader therapeutic capability and potentially circumventing the challenges posed by antibiotic resistance.

### The conjugated NbO7LL37 and NbO13LL37 can inhibit the growth of MDR *A. baumannii*

LL37 is a 37-amino acid peptide derived from the human cathelicidin family. The peptide has broad-spectrum antimicrobial activity against bacteria, viruses, and fungi (26–28). LL37 disrupts pathogen cell membranes and bacterial biofilms, leading to cell death and contributing to immune modulation, including anti-inflammatory effects and wound healing (38–40). As part of the innate immune system, LL37 plays a vital role in defending against infections. Despite its potent antimicrobial and immunomodulatory effects, LL37 can have low affinity for some targets due to its small size and electrostatic interactions with microbial membranes. This reduced affinity may limit its effectiveness in certain contexts. Additionally, LL37 can interact with albumin, the most abundant serum protein, hindering its ability to interact with pathogenic targets (41). In this study, two tigecycline-resistant *A. baumannii* clinical isolates, R422 and H222, obtained from sputum and blood samples of infected patients at Vajira Hospital, Thailand, were selected (8). These two resistant strains share highly similar amino acid sequences and three-dimensional structures with AbOmpA from *A. baumannii* strain 19606, which was used in the initial yeast display selection of Nbs (Fig. S1). Therefore, R422 and H222 were employed to evaluate the efficacy of Nb and LL37-conjugated Nb treatments in this experiment.

**Fig 8:**
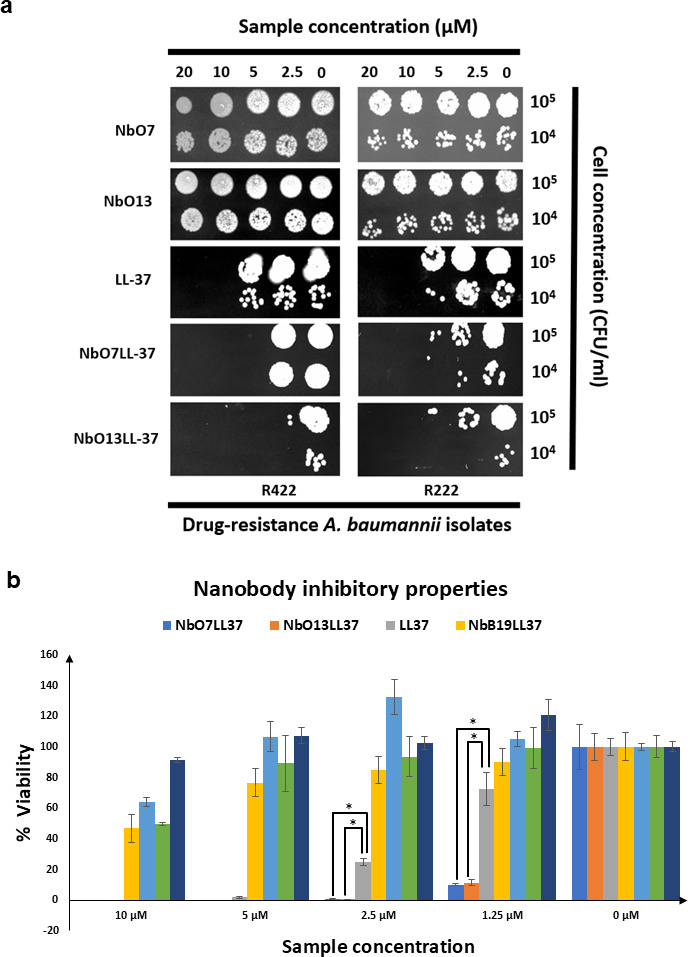
Dose-dependent antibacterial activity of NbO7LL37 and NbO13LL37 against MDR *A. baumannii.* (**a**) Inhibition of the interaction between NT-AbOmpA-specific Nbs and LL37-conjugated Nbs by a spot plate assay. (**b**) The killing efficiency of NbO7, NbO13, LL37, and their LL37-conjugated Nbs (NbO7LL37 and NbO13LL37) was assessed using a total plate count assay on tigecycline-resistant *A. baumannii* strains R422 and H222. NbO7LL37 and NbO13LL37. Data are the averages of three biological replicates and are shown as mean ± standard deviation. Statistical significance was determined using one-way ANOVA to indicate statistically significant differences (*P < 0.05).

To compare their effects against *Acinetobacter* species, the bacterial cells were treated with different concentrations of NbO7, NbO13, LL37, NbO7LL37, and NbO13LL37. The incubated cells were spotted on Mueller-Hinton agar plates. After incubation, the growth inhibition zones were measured to assess the effectiveness of each treatment. The results showed that NbO7LL37 and NbO13LL37 demonstrated significantly higher antibacterial activity against *A. baumannii* cells than NbO7, NbO13, and LL37 alone. Specifically, NbO7LL37 and NbO13LL37 demonstrated greater inhibition (Fig. 8a), suggesting that the combination of these conjugated agents enhanced their ability to disrupt bacterial growth. These findings highlight the potential of combining NbO7 and NbO13 with LL37 as an effective strategy against *A. baumannii*, a pathogen known for its resistance to multiple antibiotics. In addition to the growth inhibition assay shown in Fig. 6, the antibacterial activity of NbO7LL37 and NbO13LL37 was further evaluated using a total plate count assay. In this experiment, MDR *A. baumannii* strains R422 and H222 were incubated with varying concentrations of NbO7, NbO13, NbO7LL37, and NbO13LL37 (Fig. 8b). Consistent with the inhibition zone results, NbO7LL37 and NbO13LL37 were able to suppress bacterial growth by more than 90% at a concentration of 1.25 µM, whereas LL37 alone exhibited less than 30% killing efficiency. Furthermore, complete bacterial inhibition was achieved by both NbO7LL37 and NbO13LL37 at concentrations of 2.5 µM and above, demonstrating their potent and dose-dependent antimicrobial activity against these tigecycline-resistant *A. baumannii* isolates. However, although NbB19LL37, which is not an AbOmpA-specific Nb, also exhibited a certain degree of bacterial growth inhibition, this effect was likely due to the flexible GS linker (GGGGS)_7_ connecting the Nb and LL37 domains. The linker’s susceptibility to cleavage may have released a small fraction of free LL37 molecules, thereby contributing to the minor antibacterial activity observed against *A. baumannii*. The synergistic effect observed in the combination treatment may offer a promising approach to overcoming antibiotic resistance and improving therapeutic outcomes in infections caused by this MDR pathogen.

### Matrix-assisted laser desorption/ionization time-of-flight mass spectrometry (MALDI-TOF MS) analysis of Nb specificity toward native AbOmpA

The objective of this experiment was to determine the specificity of Nbs NbO7 and NbO13 toward the native AbOmpA protein. Whole-cell lysates of the tigecycline-resistant *A. baumannii* strain R422 were incubated with NbO7 to allow binding to surface-exposed AbOmpA. Following incubation, proteins were separated by SDS-PAGE and transferred onto membranes for Western blot analysis. Protein bands specifically recognized by NbO7 and NbO13 were excised from the SDS-PAGE gel for subsequent identification using MALDI-TOF MS (Fig. S2).

MALDI-TOF MS is a proteomic technique that ionizes peptides generated from protein digestion and measures their mass-to-charge ratios (m/z) to generate peptide mass fingerprints. These fingerprints are then matched against protein databases to identify the protein and evaluate peptide coverage, providing both qualitative and quantitative information regarding protein identity. Analysis of the excised bands revealed that the proteins specifically recognized by both NbO7 and NbO13 corresponded to Omp38 of *A. baumannii*, which is identical to the well-characterized OmpA protein. For NbO7, the MALDI-TOF MS analysis yielded a score of 1,273 with sequence coverage of 42%, while NbO13 showed a score of 376 with sequence coverage of 38% (Fig. S3 and S4). These results confirm that both Nbs selectively bind to the native OmpA protein on *A. baumannii*, validating the specificity observed in the ELISA results. Overall, this study demonstrates that NbO7 and NbO13 retain their target specificity in the context of native bacterial proteins and highlights the utility of MALDI-TOF MS as a powerful tool for confirming Nb-antigen interactions in complex cellular extracts.

### Specific recognition of *A. baumannii* surface antigens by Nbs and LL37 conjugates via whole-cell ELISA

**Fig 9:**
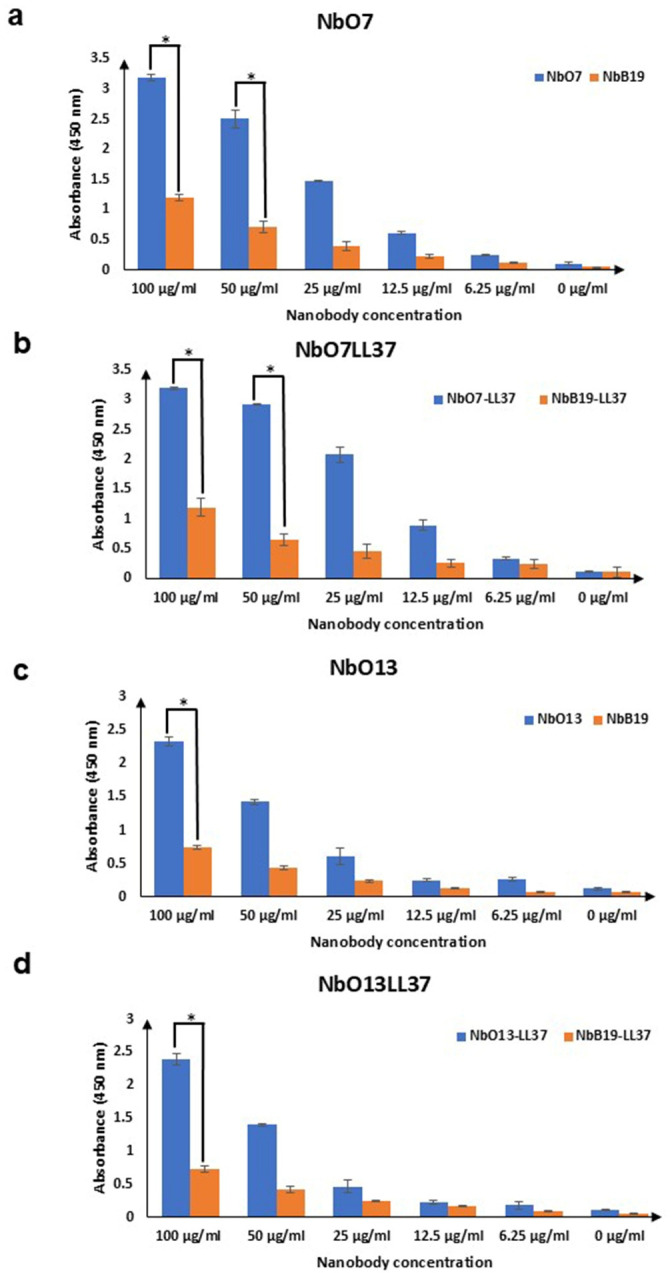
Whole-cell ELISA demonstrates high specificity of NbO7, NbO13, and their LL37 conjugates toward MDR *A. baumannii*. (**a–d**) Binding of NbO7, NbO7LL37, NbO13, and NbO13LL37 to MDR *A. baumannii* cells immobilized on 96-well plates was assessed by whole-cell ELISA. Data are the averages of three biological replicates and are shown as mean ± standard deviation. Statistical significance was determined using Student’s *t*-test to indicate statistically significant differences (*P < 0.05).

The whole-cell ELISA assay was performed to evaluate the binding specificity of selected Nbs and LL37-conjugated Nbs against *A. baumannii* R422 immobilized on 96-well plates. The results demonstrated that NbO7, NbO13, NbO7LL37, and NbO13LL37 exhibited strong binding signals to the bacterial cells, indicating effective recognition of surface-exposed antigens on intact *A. baumannii*. In contrast, NbB19 and NbB19LL37 showed markedly lower binding activity, suggesting minimal interaction with other non-specific whole-cell surface proteins (Fig. 9). These observations are consistent with previous ELISA data performed using purified NT-AbOmpA and FL-AbOmpA, confirming that Nbs targeting specific epitopes on AbOmpA can effectively recognize the protein in its native context on the bacterial surface. The enhanced binding observed with the LL37-conjugated Nbs highlights the functional advantage of fusing the AMP to NT-AbOmpA-specific Nbs. The conjugation appears to improve the targeting of LL37 to the AbOmpA protein on the bacterial cell surface, thereby increasing its local concentration and potential antimicrobial activity. This result supports the concept that Nb-mediated delivery can enhance the specificity of AMPs, enabling precise engagement with surface-exposed virulence factors while minimizing off-target interactions. Furthermore, the clear difference in binding efficiency between NbO7/NbO13 and NbB19 variants underscores the importance of selecting Nbs with high specificity toward relevant epitopes. NT-AbOmpA-specific Nbs, such as NbO7 and NbO13, likely recognize conserved epitopes within the N-terminal domain of AbOmpA that are accessible on the cell surface. These epitopes are presumably critical for mediating the observed strong and selective binding. The poor binding of NbB19 and its LL37 conjugate suggests that either the targeted epitopes are less accessible or the Nb affinity is insufficient to support robust interaction with the native protein in its cellular context.

## DISCUSSION

Due to its MDR mechanisms, the nosocomial gram-negative pathogen MDR *A. baumannii* is an emerging threat to global health, especially in hospital intensive care units (ICUs) (4). This bacterium is normally resistant to virtually all antibiotics, including carbapenems, cephalosporins, chloramphenicol, trimethoprim, aminoglycosides, colistin, and tigecycline, making infections difficult to treat and leading to increased mortality after infection (5, 6). AbOmpA is a protein that plays a crucial role in maintaining the integrity of the outer membrane, thereby facilitating adhesion to host cells and promoting bacterial survival in hostile environments (14, 32). Additionally, many features make AbOmpA a convenient target for combating *A. baumannii*. Since it is produced at every stage of *A. baumannii*’s life cycle, AbOmpA can be targeted at any time. The protein is localized on the bacterial membrane, allowing it to be easily recognized by the immune response (17). Finally, AbOmpA is highly immunogenic, triggering the host’s adaptive immune response (15, 18). Here, we applied six rounds of MACS-based yeast display biopanning to isolate two clones of NT-AbOmpA-specific Nbs. These Nbs demonstrated specific binding activity to NT-AbOmpA and thus could be employed for further characterization and therapeutic development. Based on the predictions from molecular docking, we hypothesized that the long, protruding CDR3s of both NbO7 and NbO13 may bind to sites located on the conservative extracellular flexible loops of NT-AbOmpA. Future epitope mapping will be necessary to confirm the specific location and binding mechanism. The success of this screening approach underscores the potential of using Nbs as a platform for targeting critical bacterial virulence factors. Given their small size, stability, and ease of production, these Nbs have robust potential for therapeutic application against *A. baumannii*. Conjugation of NbO7 and NbO13 with LL37 enhanced antimicrobial activity against the two clinical isolates of MDR *A. baumannii*. The results showed that both NbO7LL37 and NbO13LL37 exhibited higher antibacterial activity compared to Nbs and LL37 alone. In particular, NbO7LL37 and NbO13LL37 demonstrated superior inhibition, suggesting that conjugating these agents with LL37 significantly enhanced their antimicrobial effects. This combination strategy led to a more potent disruption of bacterial growth, highlighting the synergistic effect of the conjugated species. Furthermore, this synergistic approach not only strengthens the efficacy of the treatments for *A. baumannii* but also offers a more effective alternative to traditional monotherapy. According to previous reports, due to their unique structure, Nbs can easily bind to specific targets on bacterial cells, such as proteins or antigens on the bacterial surface. Nbs can act through several mechanisms, including interfering with the bacteria’s ability to function or replicate (42). By binding to bacterial toxins or surface proteins, Nbs can prevent the bacteria from adhering to host cells or releasing harmful substances, such as the fimbriae-specific Nb K609, which prevents fimbriae activity, leading to the interruption of gram-negative bacteria binding to the host cell surface (43). NbFedF6, NbFedF7, and NbFedF8 were able to neutralize FedF and ETECF18, the fibronectin-binding protein F of enterotoxigenic *E. coli*, hindering bacterial attachment and colonization (44). Furthermore, Nbs can inhibit key bacterial processes such as biofilm formation, a common factor in bacterial antibiotic resistance. For example, in 2015, Moayeri and his coworkers identified two VHH classes, JIK-B8 and JKH-C7, that targeted distinct epitopes of protective antigen (PA) from alpacas immunized with the protein (45). These VHHs were combined to form a heterodimeric VHH-based neutralizing agent, VNA2-PA, that demonstrated enhanced neutralizing potency in both *in vitro* and *in vivo* assays compared to the monomeric VHH (46). Moreover, Fioravanti et al. developed Nbs targeting the self-assembling process of the S-layer protein (Sap) that disrupted the formation of the S-layer of *Bacillus anthracis*, leading to reduced bacterial growth (47). Subcutaneous injection of these Nbs cleared anthrax infection and prevented death in a mouse model (48). As MDR *A. baumannii* continues to pose a serious threat in healthcare settings, these results provide a hopeful outlook for improving therapeutic outcomes and combating infections caused by this resilient bacterium. Continued exploration of combination therapies could pave the way for new treatments that are more effective in fighting drug-resistant pathogens.

## MATERIALS AND METHODS

### Preparation of biotinylated purified NT-AbOmpA

A DNA fragment of AbOmpA was amplified from *A. baumannii* genomic DNA of 14 clinical isolates from patients in the ICU of Vajira Hospital, Bangkok, Thailand (8), using the specific primers (AbOmpAFW: 5′-GCGATCCATATGGGCGTAACAGTTACTCCATTATT-3′ and AbOmpARW: 5′-GCGATCGTCGACTTATTGAGCTGCTGCAGGA-3′) and cloned into the pET28a plasmid between the NdeI and SalI restriction sites. This plasmid was then transformed into *E. coli* strain NEB10β (New England Biolabs [NEB], USA), creating pET28a(+)-AbOmpA.

### Selection of OmpA-targeting synthetic Nbs via MACS

A synthetic yeast display Nb library containing ~5 × 10^8^ unique sequences was used for the selection of NT-AbOmpA-specific Nbs through six rounds of MACS-based yeast display via a method adapted from McMahon et al. (49). Briefly, the yeast display Nb library stock was cultured in Yglc media (pH 4.5; 80 mM sodium citrate [pH 4.5], 6.7 g/L yeast nitrogen base without amino acids, 2% glucose, and 3.8 g/L Do mix-trp) at 30°C and 200 rpm for 48–72 h. After incubation, the enriched yeast cells were subcultured in Trp-dropout Yglc media (pH 6.0; 6.7 g/L yeast nitrogen base without amino acids, 2% galactose, and 3.8 g/L Do mix-trp) at 25°C and 220 rpm for 48–72 h to induce Nb expression on the yeast cell surface. Following 72 h of induction, the induced yeast cells were harvested via centrifugation at 3,000 × *g* and 4°C for 10 min. The harvested cells were then subjected to MACS isolation according to the EasySep cell separation protocol (STEMCELL Technologies Inc., Canada) with biotinylated NT-AbOmpA. After MACS, yeast cells containing NT-AbOmpA-targeting Nbs were immediately cultured in Yglc media (pH 4.5) at 30°C and 200 rpm for 72 h to increase the yeast cell numbers. Subsequently, yeast plasmids were extracted by incubating the yeast cell culture with zymolase enzyme (Zymo Research) at 37°C for 1 h to digest the yeast cell walls, followed by standard plasmid extraction using a commercial kit (Macherey-Nagel, Germany).

### Expression and purification of Nbs using the pET28a system

The genes encoding the isolated Nbs were PCR-amplified and inserted into the pET28a-scFv-GCN4 plasmid containing the C-terminal FLAG (50) and 6×His epitope tags between the NdeI and SalI restriction sites to create the pET28a-Nb-FLAG-His construct. This plasmid was then transformed into *E. coli* BL21(DE3) cells (NEB). Protein expression was induced by culturing the cells in 1 L of LB, adding 1 mM IPTG when the OD_600_ reached 0.6–0.7, and the culture was continued at 20°C with 200 rpm agitation overnight. After induction, the cells were harvested via centrifugation, and the soluble fractions were prepared as previously described. The samples were then analyzed by loading onto a 10% SDS-PAGE gel (TGX FastCast Acrylamide Solutions; Bio-Rad) for Western blotting according to standard protocols. The polyvinylidene difluoride membrane (Thermo Fisher Scientific) was probed with mouse anti-6×His-horseradish peroxidase (HRP) antibody (1:3,000; Abcam) to detect the Nbs. Gel images were captured using a Gel Doc EZ Gel Documentation System (Bio-Rad). For purification of NbO7 and NbO13, the cells were harvested by centrifugation at 6,000 × *g* and 4°C in His-flow buffer (20 mM Tris-HCl, 500 mM NaCl, 0.1% [vol/vol] Triton X-100, and 10 mM imidazole, pH 8.0) and sonicated on ice with a Sonifier SFX150 (Branson) at 60 s intervals, 40% amplitude, and a 50% duty cycle. The cell debris was then removed by centrifugation at 13,000 × *g* and 4°C for 20 min. The supernatant was collected and incubated with 1 mL of Ni-NTA resin (Bio-Rad) for 1 h at 4°C. The resin-supernatant mixture was loaded onto an Econo-Column Chromatography Column (Bio-Rad) and washed with four column volumes (CVs) of washing buffer (20 mM Tris-HCl, 500 mM NaCl, and 20 mM imidazole, pH 8.0). The target protein was eluted using three CVs of elution buffer (20 mM Tris-HCl, 500 mM NaCl, and 500 mM imidazole, pH 8.0). The eluted proteins were then buffer-exchanged with phosphate-buffered saline (PBS) using a 3 kDa molecular weight cutoff (MWCO) Vivaspin column (Thermo Fisher Scientific). Protein purity was evaluated by SDS-PAGE and staining with InstantBlue Coomassie Protein Stain (Abcam). Finally, the concentrations of the purified Nbs were measured by absorbance at 280 nm using a NanoDrop Lite (Thermo Fisher Scientific).

### Direct binding assay by ELISA

For binding assays, purified NT-AbOmpA and FL-AbOmpA were diluted in ELISA coating buffer (0.05 M NaCO_3_, pH 9.6) to a final concentration of 10 µg/mL, and 50 µL of the mixture was coated onto 96-well plates (Corning) and incubated overnight at 4°C. The plate was then blocked with 5% non-fat milk in TBS overnight at 4°C. After the plates were washed with PBST (8.1 mM Na_2_HPO_4_, 1.5 mM KH_2_PO_4_, 137 mM NaCl, and 2.7 mM KCl containing 0.1% Tween-20), 50 µL/well of either lysate (at various concentrations, with total protein measured by a Quick Start Bradford assay, Bio-Rad) or purified Nbs (2.5, 5, 10, 20, 40, and 80 µg/mL) diluted in PBS was added and incubated for 1 h at RT. After washing with PBST, 50 µL/well of anti-FLAG-HRP antibody (1:3,000; Abcam) in PBS was added and incubated for 1 h at RT. After final washes with PBST, 100 µL/well of the 1-step TMB ELISA substrate solution (Thermo Fisher Scientific) was added. Finally, 100 µL/well of 1 M H_2_SO_4_ was added to quench the reaction, and the absorbance of the wells was measured at 450 nm (Infinite M200; Tecan Austria GmbH).

### Protein extraction and identification from tigecycline-resistant *A. baumannii* R422 by Western blot analysis and MALDI-TOF MS

The tigecycline-resistant *A. baumannii* strain R422 was cultured in tryptic soy broth (TSB) at 37°C overnight. The bacterial culture was centrifuged at 6,000 × *g* for 10 min at 25°C, and the cell pellet was resuspended in PBS containing 1% SDS. The suspension was boiled at 100°C and then centrifuged at 12,000 × *g* for 10 min at 25°C. The supernatant was collected, and the protein concentration was determined using the Bradford assay. A total of 10 µg of extracted protein was subjected to SDS-polyacrylamide gel electrophoresis (SDS-PAGE), followed by transfer onto a polyvinylidene difluoride membrane. The membrane was probed with NbO7 and NbO13, followed by incubation with rabbit anti-FLAG-HRP conjugate (1:3,000; Abcam). Gel images were captured using a Gel Doc EZ Documentation System (Bio-Rad). Protein bands of interest were excised from the gel and subjected to identification by MALDI-TOF MS.

### Expression and characterization of NT-AbOmpA, FL-AbOmpA, NbO7LL37, and NbO13LL37 in an *E. coli* expression system

The pET28a-NbO7LL37-FLAG-His plasmid was synthesized with codon optimization for *E. coli* expression by GenScript, Inc. Then, the gene encoding NbO7 was replaced by the NbO13 gene via the NdeI and BamHI restriction sites in the pET28a-NbO7LL37-FLAG-His plasmid, generating the pET28a-NbO13LL37-FLAG-His, while NbO7LL37 was replaced by the FL-AbOmpA gene via NdeI and speI restriction sites to create pET28a-FL-AbOmpA-FLAG-His. Protein expression analysis was conducted following the same protocol as described for FL-AbOmpA NbO7LL37 and NbO13LL37. A procedure adapted from Tantiwiwat et al. (51) was employed to purify and refold NbO7 and NbO13 conjugated with LL37. *E. coli* BL21(DE3) cells were subcultured into fresh LB medium, and protein expression was induced at 20°C overnight with 1 mM IPTG. After harvesting 200 mL of the culture, the cells were resuspended (1 g wet cell weight per 4 mL) in Base buffer (20 mM Tris-HCl, 6 M urea, 500 mM NaCl, 5 mM β-mercaptoethanol, and 10% vol/vol glycerol). Sonication was performed on ice using a Sonifier SFX150 (Branson) at 45% amplitude and 50% duty cycle for 70 cycles of 30 seconds each. Refolding buffer (20 mM Tris-HCl, 500 mM NaCl, 55 mM glucose, 2 mM reduced glutathione [GSH], 0.2 mM oxidized glutathione [GSSG], and 20% [vol/vol] glycerol) supplemented with 2 M urea was then added to the lysate at a 1:9 (vol/vol) ratio and incubated on a rocking shaker for 1 h at 20°C. Afterward, the precipitate was removed by centrifugation at 4°C, 10,000 × *g* for 30 min. The supernatant was applied to a HisTrap column with an Econo-Column (GE Healthcare) at room temperature. Refolding of the protein trapped on the HisTrap column was achieved by washing with two CVs of refolding buffer containing decreasing concentrations of urea (6, 4, 2, 1, and 0 M). Non-specific proteins were washed off using 10 CVs of refolding buffer containing 50 mM imidazole. The target protein was eluted with 250 mM imidazole. Finally, imidazole was removed, and the buffer was exchanged with TBS using a 10 kDa MWCO centrifugal concentrator.

### Evaluation of refolded FL-AbOmpA folding through functional cytotoxicity using MTT assay

L929 fibroblast cells were cultured in DMEM high-glucose medium supplemented with 10% FBS and 1% penicillin-streptomycin at 37°C in a humidified 5% CO₂ incubator. Cells were maintained in T-25 flasks and seeded at a density of 20,000 cells per well in 96-well plates. After 24 h of incubation to allow cell attachment, the medium was replaced with fresh DMEM containing refolded or denatured FL-AbOmpA at final concentrations of 10, 2.5, 0.6125, 0.15, and 0 µM. The denatured FL-AbOmpA was prepared by boiling the protein for 10 min prior to use. Following 24 h incubation, cell viability was assessed using the MTT assay. Briefly, 10 µL of MTT solution (5 mg/mL) was added to each well and incubated for 4 h at 37°C. The supernatant was then removed, and the resulting formazan crystals were dissolved in 100 µL of DMSO. Absorbance was measured at 570 nm using a microplate reader, and cell viability was calculated relative to untreated controls to evaluate the biological activity and correct folding of the refolded FL-AbOmpA protein.

### Specificity testing of Nbs and LL37-conjugated Nbs using whole-cell ELISA

The tigecycline-resistant *A. baumannii* strain R422 was cultured in TSB at 37°C overnight. After overnight growth, bacterial cells were harvested and adjusted to a concentration of approximately 1 × 10⁶ cells per well. The cells were immobilized on 96-well microtiter plates by coating with ELISA coating buffer (0.05 M NaHCO₃, pH 9.6) and incubating overnight at 4°C. Following immobilization, the bacterial cells were inactivated by incubation with 4% paraformaldehyde for 15 min at room temperature. Wells were then washed three times with PBS and blocked with ELISA blocking buffer (5% skim milk in PBS) for 1 h at room temperature to prevent non-specific binding. After blocking, the wells were incubated with the test Nbs NbO7, NbO13, NbO7LL37, and NbO13LL37 at appropriate concentrations. Following primary incubation, wells were washed with PBS and incubated with anti-FLAG-HRP conjugate to detect bound Nbs. The enzymatic signal was developed and detected using a Gel Doc EZ Gel Documentation System (Bio-Rad), and the binding intensity was quantified to assess the specificity of each Nb and LL37-conjugated Nb toward *A. baumannii* R422 cells.

### Antimicrobial activity assay against tigecycline-resistant *A. baumannii* clinical isolates

Two tigecycline-resistant *A. baumannii* clinical isolates, R422 and H222, obtained from patients at Vajira Hospital, Thailand, were used in this study (8). The bacterial strains were cultured in TSB at 37°C overnight. The bacterial suspensions were then adjusted to final concentrations of approximately 10⁵ and 10⁴ CFU/mL for strains R422 and H222, respectively. The diluted bacterial suspensions were mixed with test samples (NbO7, NbO13, LL37, NbO7LL37, and NbO13LL37) at final concentrations of 0, 2.5, 5, 10, and 20 µM, respectively. The mixtures were incubated at 37°C for 1 h. Following incubation, aliquots of each mixture were spotted onto TSB agar plates and incubated at 37°C overnight. The survival of *A. baumannii* strains R422 and H222 was then evaluated by observing bacterial growth on the tryptic soy agar (TSA) agar plates.

Bacterial suspensions adjusted to approximately 10⁴ CFU/mL were then used for the inhibition assay. The tigecycline-resistant *A. baumannii* isolates R422 and H222 were incubated with the test samples (NbO7, NbO13, NbB19, LL37, NbO7LL37, NbO13LL37, and NbB19LL37) at final concentrations of 0, 1.25, 2.5, 5, and 10 µM. The mixtures were incubated at 37°C for 1 h. After incubation, the bacterial suspensions were serially diluted to appropriate concentrations and spread onto TSA plates. The plates were then incubated at 37°C overnight. The number of surviving bacterial colonies was counted to determine the inhibitory effect of each test sample.

### Molecular modeling and protein 3D structure analysis of NbO7 and NbO13

All Nb sequences were uploaded to the SWISS-MODEL server (https://swissmodel.expasy.org) to create 3D Nb structures using model P56963.1.A as the 3D protein structure template for 3D structure prediction. The PyMOL modeling program (version 2.5) was used for molecular labeling. The interactions between NbTO7 and NbO13 with NT-AbOmpA were simulated using the ClusPro 2.0 server (https://cluspro.bu.edu/login.php), and the Discovery Studio 2.0 program was used to visualize the molecular interactions between the Nbs and their specific targets.

## Data Availability

The data sets generated and/or analyzed during the current study are deposited in GenBank under accession numbers PX917034 and PX917035. They are expected to be available 27 January 2027. Prior to that date, please contact the corresponding author to access the data, which cannot be more widely released because of requirements of the funding agency.

## SUPPLEMENTAL MATERIAL

Supplemental material msphere.00779-25-s0001.pdf Figures S1-S4.

ASM does not own the copyrights to Supplemental Material that may be linked to, or accessed through, an article. The authors have granted ASM a non-exclusive, world-wide license to publish the Supplemental Material files. Please contact the corresponding author directly for reuse.
