# Supplementary material for: Development of nanobody-conjugated LL37 for synergistic therapy against MDR Acinetobacter baumannii
Source: mSphere. 2026 Feb 6;11(3):e00779-25. doi: 10.1128/msphere.00779-25 (PMC13037418; doi:10.1128/msphere.00779-25)
Supplement: Supplemental material — Figures S1-S4. [file msphere.00779-25-s0001.pdf]

Supplementary data

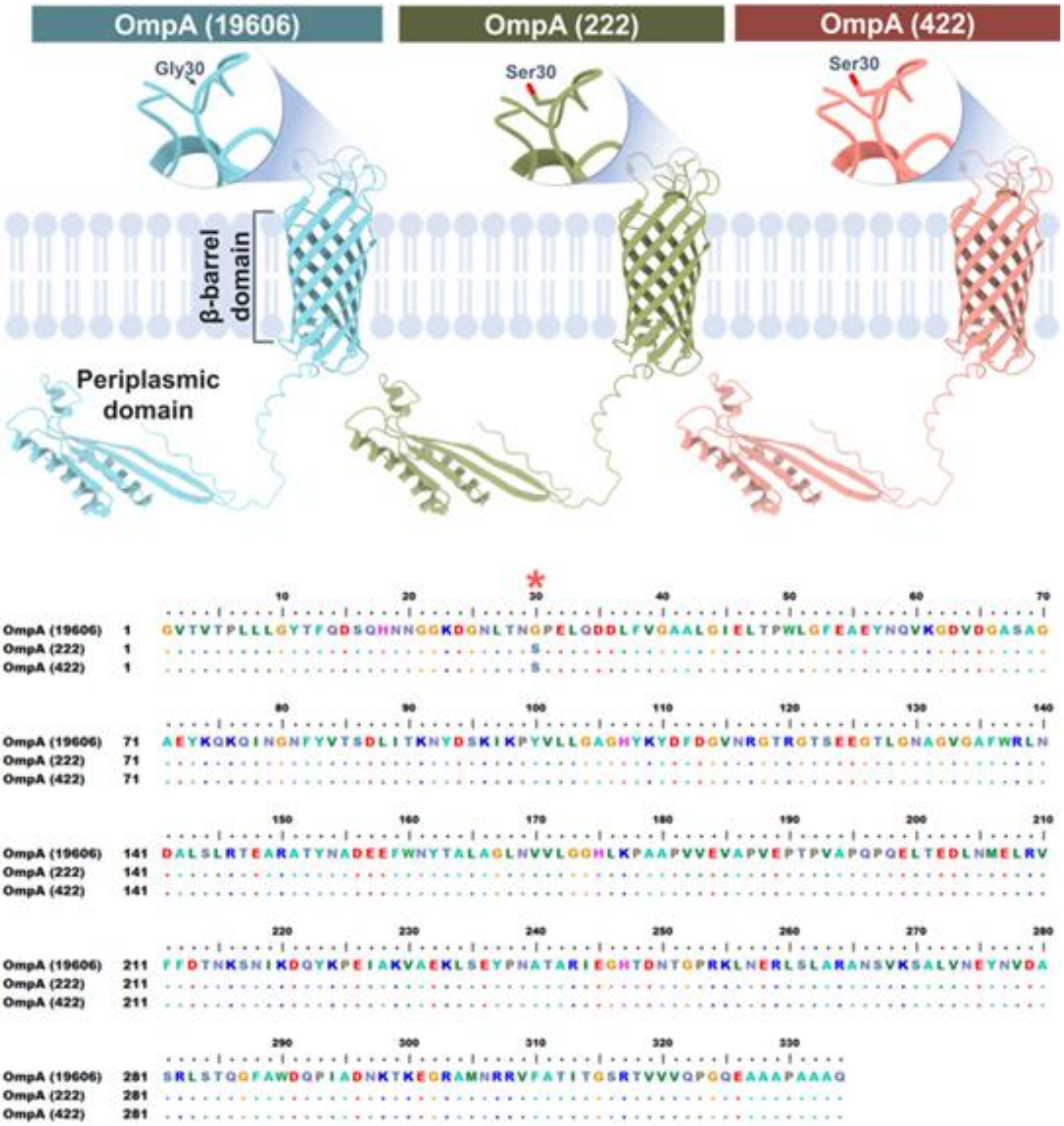

Supplementary Fig. 1 : The upper figure represents three AbOmpA 3D structures from non-drug-resistant strains of *A. baumannii 19606*, and the other two AbOmpA 3D structures of multi drug-resistant *A. baumanii* isolated from a hospital in Thailand. The lower figure shows a comparison of amino acid residues of AbOmpA among three *A. baumanii* isolates.

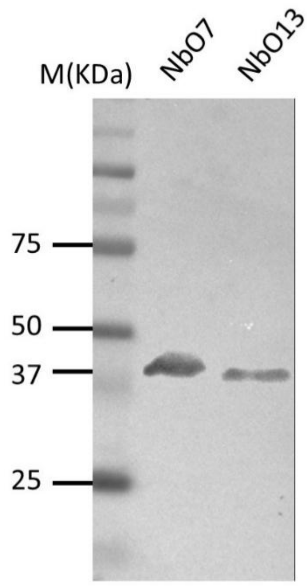

Supplementary Fig. 2 : Specificity analysis of NbO7 and NbO13 by Western blot. Whole-cell lysates of tigecycline-resistant *A. baumannii* R422 were probed with NbO7 and NbO13. Protein bands observed above 37 kDa were excised and subsequently analyzed by MALDI-TOF mass spectrometry to identify the bound proteins.

MATRIX SCIENCE **MASCOT Search Results**

Protein View: A0A009IV33_ACIB9

Outer membrane protein omp38 OS=Acinetobacter baumannii (strain 1295743) OX=1310613 GN=omp38 PE=4 SV=1

**Database:**	uniprotkb_Acinetobacter_baumannii_2025_07_29
**Score:**	1273
**Monoisotopic mass (M_r):**	38397
**Calculated pI:**	5.32

Sequence similarity is available as an NCBI BLAST search of A0A009IV33_ACIB9 against nr.

Search parameters

**MS data file:**	1.mgf
**Enzyme:**	Trypsin/P: cuts C-term side of KR.
**Fixed modifications:**	Carbamidomethyl (C)
**Variable modifications:**	Oxidation (M)

Protein sequence coverage: 42%

Matched peptides shown in ***bold red***.

```
  1 MKLSRIALAT MLVAAPLAAA NAGVTVTPLL LGYTFQDSQH NNGGKDGNLT
 51 NGPELQDDLF VGAALGIELT PWLGFEAEYN QVKGDVDGAS AGAEYKQKQI
101 NGNFYVTSDL ITKNYDSKIK PYVLLGAGHY KYDFDGVNRG TRGTSEEGTL
151 GNAGVGAFWR LNDALSLRTE ARATYNADEE FWNYTALAGL NVVLGGHLKP
201 AAPVVEVAPV EPTPVAPQPQ ELTEDLNMEL RVFFDTNKSN IKDQYKPEIA
251 KVAEKLSEYP NATARIEGHT DNTGPRKLNE RLSLARANSV KSALVNEYNV
301 DASRLSTQGF AWDQPIADNK TKEGRAMNRR VFATITGSRT VVVQPGQEAA
351 APAAAQ
```

Supplementary Fig. 3 : MALDI-TOF mass spectrometry analysis was performed on protein bands excised from SDS-PAGE gels following Western blotting of whole-cell proteins from *A. baumannii* R422 after incubation with NbO7.

MATRIX SCIENCE MASCOT Search Results

Protein View: A0A009IV33_ACIB9

Outer membrane protein omp38 OS=Acinetobacter baumannii (strain 1295743) OX=1310613 GN=omp38 PE=4 SV=1

**Database:**	uniprotkb_Acinetobacter_baumannii_2025_07_29
**Score:**	236
**Monoisotopic mass (M_r):**	38397
**Calculated pI:**	5.32

Sequence similarity is available as an NCBI BLAST search of A0A009IV33_ACIB9 against nr.

Search parameters

**MS data file:**	2.mgf
**Enzyme:**	Trypsin/P: cuts C-term side of KR.
**Fixed modifications:**	Carbamidomethyl (C)
**Variable modifications:**	Oxidation (M)

Protein sequence coverage: 38%

Matched peptides shown in ***bold red***.

```
  1 MKLSRIALAT MLVAAPLAAA NAGVTVTPLL LGYTFQDSQH NNGGKDGNLT
 51 NGPELQDDLF VGAALGIELT PNLGFEAEYN QVKGDVDGAS AGAEYKQKQI
101 NGNFYVTSDL ITKNYDSKIK PYVLLGAGHY KYDFDGVNRG TRGTSEEGTL
151 GNAGVGAFWR LNDALSLRTE ARATYNADEE FWNYTALAGL NVVLGGHLKP
201 AAPVVEVAPV EPTPVAPQPQ ELTEDLNMEL RVFFDTNKSN IKDQYKPEIA
251 KVAEKLSEYP NATARIEGHT DNTGPRKLNE RLSLARANSV KSALVNEYNV
301 DASRLSTQGF AWDQPIADNK TKEGRAMNRR VFATITGSRT VVVQPGQEAA
351 APAAAQ
```

Supplementary Fig. 4: MALDI-TOF mass spectrometry analysis was performed on protein bands excised from SDS-PAGE gels following Western blotting of whole-cell proteins from *A. baumannii* R422 after incubation with NbO13.
